# Metagenomic Insights into the Seasonal Distribution and Dissemination Risks of Biocide and Metal Resistance Genes in a Subtropical Coastal Ecosystem

**DOI:** 10.3390/microorganisms14071480

**Published:** 2026-07-07

**Authors:** Lihong Gan, Shiyun Fang, Hengsong Wu, Tianhao Yao, Wenjian Chen, Yusen Li, Yaoquan Han, Lei Zhou

**Affiliations:** 1 University Joint Laboratory of Guangdong Province, Hong Kong and Macao Region on Marine Bioresource Conservation and Exploitation, College of Marine Sciences, South China Agricultural University, Guangzhou 510642, China; 15766348474pp@stu.scau.edu.cn (L.G.);; 2 Key Laboratory of Aquaculture Genetic and Breeding and Healthy Aquaculture of Guangxi, Guangxi Academy of Fishery Sciences, Nanning 530021, China; 3 Engineering Research Center of Hongshui River Rare Fish Conservation, Guiping 537200, China

**Keywords:** Beibu Gulf, biocide and metal resistance genes, mobile genetic elements, seasonal variations, horizontal gene transfer

## Abstract

The widespread use of antimicrobial biocides and metals has led to the continuous accumulation of biocide and metal resistance genes (BMRGs) in the environment. The issue is of growing concern, as it reduces the efficacy of these agents and poses a potential threat to coastal ecological security. However, the extent of coastal BMRG pollution, its transmission mechanisms, and the influence of seasonal variations on its assembly remain poorly understood. In this study, metagenomic sequencing was employed to investigate BMRGs, microbiomes, and mobile genetic elements (MGEs) within the subtropical nearshore ecosystem of the Beibu Gulf during the autumn and winter seasons. A total of 33 BMRG types and 457 subtypes were detected, with higher subtype diversity in winter than in autumn (440 vs. 326 subtypes). Notably, genes resistant to multi-biocides exhibited the highest diversity, whereas those resistant to both biocides and metals were the most abundant. Co-occurrence network analysis showed that 22 of the 23 detected BMRGs in the winter network were associated with MGEs, especially transposase-related elements such as *tnpA*. Path modeling indicated that BMRG abundance was more strongly associated with bacterial community composition in autumn, whereas MGE-related variables showed stronger associations in winter. These findings suggest a pronounced seasonal shift in the underlying mechanisms shaping BMRG dynamics, with bacterial communities playing a dominant role in autumn and MGEs playing a more critical role in winter. This seasonal shift highlights the need for season-specific monitoring of BMRGs, coastal pollution control, and resistance-risk management in subtropical coastal ecosystems.

## 1. Introduction

The widespread use of antimicrobial agents in agricultural, industrial, and clinical settings has imposed continuous selective pressure on environmental microbiomes. While antibiotic resistance genes (ARGs) have been widely investigated because of their relevance to human health, resistance genes associated with biocides and metals (BMRGs) have received comparatively less attention [1,2]. This imbalance is noteworthy, as BMRGs can compromise the effectiveness of biocides and metals and may contribute to the co-selection and environmental dissemination of resistance determinants and other functional genes, thereby raising concerns regarding environmental resistance risk in coastal ecosystems [3]. Therefore, BMRGs should not be regarded simply as a subset of the broader resistome, because biocides and metals can persist in aquatic environments and impose long-term selective pressure independent of antibiotic exposure [4,5]. Moreover, BMRGs may be maintained together with ARGs or other resistance genes on shared mobile genetic platforms, thereby contributing to co-selection and multi-resistance dissemination in coastal microbiomes [3].

Biocides and metals have been widely used as antimicrobial agents for decades, imposing a substantial burden on the environment [6]. Biocides, including antiseptics and disinfectants, are extensively applied in domestic, medical, and industrial settings to control undesirable microorganisms [7,8]. Consistently, high concentrations of various biocides have been detected in wastewater and aquatic systems [9]. However, their widespread and often inappropriate use exerts strong selective pressure on microbial communities, thereby facilitating the emergence of resistant bacteria [7,10]. Similarly, agricultural intensification has promoted the widespread use of heavy metals in animal feed for disease prevention and growth promotion. This practice facilitates the emergence of heavy-metal-resistant bacteria, which subsequently release large quantities of metal resistance genes (MRGs) into the environment through feces [11]. Elevated abundances of MRGs have been widely reported in polluted aquatic systems and are often positively correlated with metal concentrations [12]. In addition, metals frequently enter marine environments through aquaculture activities, such as the use of copper, iron, and zinc in antifouling coatings and feed additives [13]. These widespread anthropogenic inputs create persistent selective pressures that favor the accumulation and maintenance of BMRGs in coastal ecosystems [14]. Notably, BMRGs are characterized by “easy to get” and “hard to lose”, which further exacerbates their risk [1].

Beyond their accumulation, the dissemination of resistance genes represents a critical ecological concern. Resistance genes proliferate not only through vertical inheritance within bacterial lineages [15], but, more importantly, through horizontal gene transfer (HGT) mediated by mobile genetic elements (MGEs) [16,17]. MGEs, such as plasmids and transposons, frequently carry multiple resistance genes simultaneously, facilitating co-resistance across microbial taxa [18]. For example, plasmids larger than 20 kb carry both BMRGs and ARGs on the same plasmid, and many of these plasmids are conjugative [19]. Consequently, the co-existence of heavy metals and antibiotics exerts dual selective pressures on microbiomes, dynamically reshaping the abundance and composition of the overall resistome [20,21]. This implies that bacterial exposure to biocides and metals not only selects for BMRGs but may also facilitate the dissemination of other functional genes through co-selection mechanisms [6,22].

In addition to bacteria, other microbial groups also contribute to resistome dynamics in marine ecosystems. Archaeal communities and viral populations can act as reservoirs and vectors for resistance genes. Seasonal studies in coastal ecosystems have shown stronger associations between archaea and ARGs during certain periods, suggesting their potential role in gene dissemination [23]. Viral communities, especially in aquaculture and microplastic biofilms, can harbor ARGs and virulence factors, thereby further facilitating horizontal gene transfer [24,25]. Environmental factors such as nitrogen levels influence microbial diversity, indirectly shaping ARG profiles in sediments [25]. Overall, the interplay among microbial communities, their associated MGEs, and environmental conditions creates a complex network governing resistome dynamics.

Despite these recognized risks, the vast majority of environmental monitoring has primarily focused on ARG contamination [2]. The dissemination dynamics of BMRGs and their underlying correlations with MGEs and microbial hosts remain largely uncharacterized. Moreover, coastal ecosystems are subject to pronounced seasonal variability, which can influence microbial community structure and gene transfer processes. However, how seasonal fluctuations affect the assembly and transmission mechanisms of BMRGs remains largely unexplored.

The Beibu Gulf is a semi-enclosed subtropical bay along the northern South China Sea. This region is affected by multiple land-based and coastal anthropogenic inputs, including riverine inputs, wastewater-related nutrient loading, aquaculture activities, and coastal development, which may introduce nutrients, metals, biocides, and other selective agents into nearshore waters [3,13,14]. The potential risk of BMRGs is environmentally relevant because biocide- and metal-resistance genes can co-occur with ARGs on bacterial chromosomes and plasmids, thereby increasing the likelihood of co-selection under non-antibiotic selective pressures [19,26]. In addition, disinfectant-resistance genes such as *qacEΔ* and *qacEΔ1* have been reported in clinically relevant Gram-negative bacteria, including members of Enterobacterales, Pseudomonas aeruginosa, and Klebsiella pneumoniae [27,28,29,30]. Collectively, these characteristics make the Beibu Gulf an ideal natural system for investigating how seasonal variation, environmental factors, microbial communities, and MGEs jointly shape the distribution and dissemination risks of BMRGs. However, comprehensive studies on the dynamics of BMRGs and their potential dissemination mechanisms in this region remain limited.

In this study, we employed metagenomic sequencing to characterize the diversity and distribution of BMRGs, MGEs, and microbial communities across autumn and winter. Specifically, we aimed to: (1) assess seasonal variation in the diversity and abundance of BMRGs; (2) explore co-occurrence relationships among BMRGs, MGEs, and microorganisms to identify potential hosts and dissemination risks; and (3) disentangle the relative roles of microbial communities, MGEs, and environmental factors in shaping BMRG assembly across seasons.

## 2. Materials and Methods

### 2.1. Sampling Sites and Sample Collection

**Figure 1:**
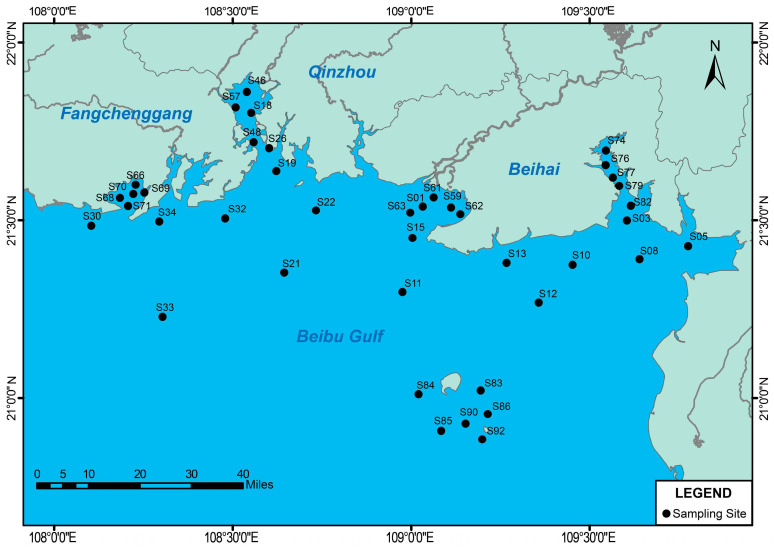
Map of the sampling stations in Beibu Gulf, China.

In this study, 40 sampling sites were selected across the coastal areas of the Guangxi Zhuang Autonomous Region and surveyed during the autumn and winter seasons (Figure 1). The sampling campaigns covered two seasonal periods, with autumn samples collected in October 2022 and winter samples obtained in January 2023, allowing us to compare BMRG patterns between autumn and winter environmental contexts. The sites were distributed across coastal areas near Fangchenggang, Qinzhou, and Beihai, and extended from nearshore bays and coastal waters to more open waters of the gulf. This spatial design was intended to capture spatial heterogeneity across different coastal settings and to reduce bias caused by sampling from a single local area. To ensure spatial representativeness, three independent spatial replicates were obtained 30 to 50 m apart along the riverbank at every station. Water samples were collected at a depth of 0.5 m and immediately filtered through 0.22-μm polycarbonate filters. The resulting filter membranes were placed in sterile cryovials, immediately immersed in liquid nitrogen, transported to the laboratory in a liquid-nitrogen tank, and then stored at −80 °C until DNA extraction.

### 2.2. Environmental Variables

The dissolved oxygen (DO), salinity, water temperature (Water_Temp) and pH were measured synchronously in situ using a multiparameter water quality sonde (YSI ProPlus, YSI Inc., Yellow Springs, OH, USA), while water transparency was determined using a Secchi disk (SunVote, Changsha, China). Total organic carbon (TOC), dissolved organic carbon (DOC), and total nitrogen (TN) were measured using an elemental analyzer (Vario EL III, Elementar Analysensysteme GmbH, Langenselbold, Germany). Active phosphate (Active_P) and active silicate (Active_Si) concentrations were accurately measured via phosphomolybdenum blue and silicomolybdenum yellow spectrophotometric methods, respectively [31]. Nitrate (NO_3_-N) and nitrite (NO_2_-N) levels were analyzed using a modified zinc-cadmium reduction method [32]. The concentration of ammonia nitrogen (NH_4_-N) was quantified utilizing the hypobromite oxidation method [33]. Ultimately, the inorganic nitrogen (Inorg-N) content was not measured directly, but mathematically derived by summing the concentrations of NO_3_-N, NO_2_-N, and NH_4_-N. Total phosphorus (TP) in water was determined according to Method 365.1 [34]. Gravimetric analysis is a quantitative measurement method based on the solid mass of the analyte, using which suspended solids (SS) were measured [35]. Chlorophyll-a (Chl_a) was measured according to the standards required by the laboratory [36].

### 2.3. DNA Extraction and Metagenomics Sequencing

To minimize technical bias, all samples were processed using identical DNA extraction, library preparation, sequencing, and quality-control procedures. The FastDNA Spin Kit for Soil (MP Biomedicals, Santa Ana, CA, USA) was used to extract total microbial DNA. Before library preparation and sequencing, DNA quality was assessed by the commercial sequencing provider according to its standard workflow. Nucleic acid purity and concentration were quantified using a NanoDrop One spectrophotometer and a Qubit 4.0 fluorometer (Thermo Fisher Scientific, Waltham, MA, USA), respectively. For each sample, library construction was initiated with 1 μg of genomic DNA. Sequencing libraries were generated utilizing the VAHTS^®^ Universal Plus DNA Library Prep Kit for Illumina V2 (Vazyme Biotech Co., Ltd., Nanjing, China) according to the manufacturer’s instructions. Metagenomic sequencing was subsequently performed on the Illumina NovaSeq 6000 platform (Illumina, San Diego, CA, USA) using a 150 bp paired-end read strategy, yielding an average of 15 Gbp of raw data per sample and a cumulative metagenomic output of approximately 1.18 Tbp.

### 2.4. Annotation of BMRGs, MGEs and Microbiome

To ensure analytical consistency and minimize bioinformatic bias, raw sequencing reads from all samples were processed using the same workflow and parameters, including stringent quality filtering using fastp v0.23.2 [37] and contaminant removal with bowtie2 v2.5.1 [38] under default parameters. The taxonomic composition of the microbial communities, including bacteria, archaea, and viruses, was profiled using Kraken2 v2.1.2 [39], with species-level quantification further refined by Bracken v2.5 [40]. To identify BMRGs and MGEs, clean reads from the metagenomic datasets were aligned to the BacMet database (version 2.0) [6], and the MobileGeneticElement database [41], respectively, through ARG-OAP v3.0 [42] with customized reference databases. Annotations followed the recommended ARG-OAP v3.0 thresholds, including an E-value threshold of 1 × 10^−7^, sequence identity of at least 80%, and a hit length ratio of at least 75%. The BacMet database was used for BMRG annotation, whereas the MobileGeneticElement database was used to identify MGEs, including plasmids, integrons, transposons, and insertion sequences. For abundance normalization, 16S rRNA gene copy numbers were estimated from the metagenomic reads using the ARG-OAP v3.0 normalization workflow. The abundance of each BMRG subtype was then normalized to the corresponding 16S rRNA gene copy number in the same sample, and the normalized abundance was reported as copies per 16S rRNA gene.

### 2.5. Statistical Analysis and Data Visualization

Statistical analyses were performed using R version 4.5.2. BMRG diversity was represented by the number of detected BMRG types or subtypes, whereas BMRG abundance was based on normalized abundance values expressed as copies per 16S rRNA gene. Because most BMRG abundance and diversity data did not follow a normal distribution, differences between autumn and winter samples were assessed using the Wilcoxon rank-sum test, with *p* < 0.05 considered statistically significant. Linear regression analysis based on Bray–Curtis distances was conducted to evaluate the consistency among the compositions of BMRGs, MGEs, and microbial communities.

To characterize the associations among MGEs, microbial communities, and BMRGs, Spearman rank correlations were used to construct co-occurrence networks. Correlation analyses were performed using BMRG subtypes, MGE subtypes, and microbial families detected in at least 60% of the samples, with statistically significant associations defined by Spearman correlation coefficients of *r* > 0.8 and *p* < 0.01. Network visualization was conducted in Gephi 0.10.1, and network topology was explored using the Fruchterman-Reingold layout [43]. Mantel tests were performed to evaluate correlations between environmental variables and the community structures of BMRGs and MGEs based on distance matrices. Spearman rank correlation analysis was further used to examine relationships between individual environmental variables and the abundance of the top 10 BMRG types. Variance partitioning analysis (VPA) was used to quantify the independent and shared contributions of MGEs and microbial communities to BMRG compositional variation. Partial least squares path modeling (PLS-PM) was implemented using the “plspm” package to evaluate potential direct and indirect pathways linking environmental factors, microbial communities, MGEs, and BMRGs. In this study, coordinate scores from PCoA were used as manifest variables to represent the latent constructs of microbial communities, MGEs, and BMRGs. The selection of specific PCoA axes (PCoA1 and/or PCoA2) was guided by their statistical significance and correlation strength with the target variables to ensure maximum explanatory power. Data visualization was primarily performed using the “ggplot2” and “pheatmap” packages.

## 3. Results

### 3.1. Distribution of BMRGs and Their Spatiotemporal Difference in Beibu Gulf

**Figure 2:**
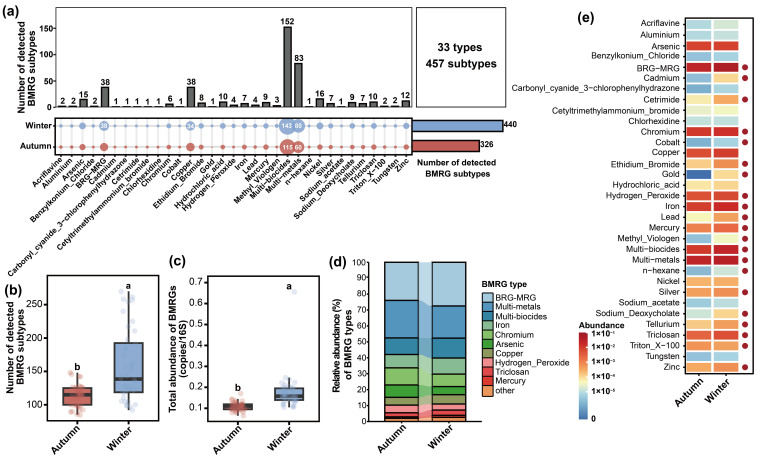
Seasonal distribution patterns of BMRGs. (**a**) Comparison of BMRG types identified across seasons. (**b**) Number of BMRG subtypes detected in autumn and winter. (**c**) Total BMRG abundance in different seasons. Each lowercase letter indicates a statistically significant variation between seasons based on the Wilcoxon test (*p* < 0.05). (**d**) Relative proportions of different BMRG types within the total BMRG abundance. (**e**) Shifts in BMRG type abundance across seasons. Red dots denote significant seasonal differences, Wilcoxon test (*p* < 0.05).

A total of 33 BMRG types and 457 subtypes were identified across all samples, with each sample containing 85 to 270 subtypes. Among them, genes conferring resistance to multiple biocides were the most diverse group (152 subtypes), followed by multi-metal resistance genes (83 subtypes), copper (Cu) resistance genes (38 subtypes), and genes associated with both biocide and metal resistance (BRG-MRGs, 38 subtypes) (Figure 2a). Seasonal analysis identified 326 BMRG subtypes in autumn samples, and 440 subtypes in winter samples, indicating increased BMRG diversity in winter (*p* < 0.05, Figure 2b). The number of multi-biocide resistance genes in winter (143 subtypes) was significantly higher than that in autumn (115 subtypes), and the number of multi-metal resistance genes in winter (80 subtypes) was also higher than that in autumn (60 subtypes) (Figure 2a). The highest numbers of BMRGs were detected at site AuS77 in autumn (148 subtypes) and site WiS19 in winter (270 subtypes) (Figure S1). Although multi-biocide resistance genes represented the most diverse BMRG category, the number of detected subtypes varied considerably among samples, ranging from 19 to 57 in autumn and increasing from 25 to 89 in winter (Figure S1). In contrast, multi-metal resistance genes displayed relatively stable occurrence patterns, although subtype diversity increased markedly in nearly all winter samples compared with autumn (Figure S1). These results describe the diversity pattern of BMRGs based mainly on the numbers of detected types and subtypes.

The abundance pattern showed a similar seasonal trend but differed in the dominant BMRG categories. Similar to the pattern observed in BMRG diversity, BMRG abundance was substantially higher in winter samples compared with autumn samples (*p* < 0.05, Figure 2c). The most prevalent BMRG type was genes conferring resistance to both biocides and metals (BRG-MRGs), followed by multi-metal, multi-biocides, iron, and chromium resistance genes (Figure 2d). This differed from the subtype-diversity pattern, where multi-biocide resistance genes were the most diverse category. In addition to the dominant BMRG categories, several other BMRG types also exhibited significant seasonal differences. A considerable proportion of BMRG types displayed higher abundance in winter compared with autumn, including resistance genes for metals (cadmium, chromium, cobalt, gold, iron, lead, mercury, silver, tellurium, and zinc), biocides (cetrimide, ethidium bromide, hydrogen peroxide, methyl viologen, sodium deoxycholate, and triclosan), as well as multi-resistance genes (BRG-MRGs, multi-biocides, and multi-metals) (*p* < 0.05, Figure 2e). Conversely, genes conferring resistance to Triton X-100 were significantly more abundant in autumn than in winter (*p* < 0.05, Figure 2e). At the subtype level, *ruvB* (multi-metals) was the most abundant BMRG, followed by *recG* (multi-metals), *acn* (iron), and *actR* (BRG-MRGs) resistance genes (Figure S2).

### 3.2. Seasonal Variation in Microbial and MGE Contributions to BMRG Dynamics

**Figure 3:**
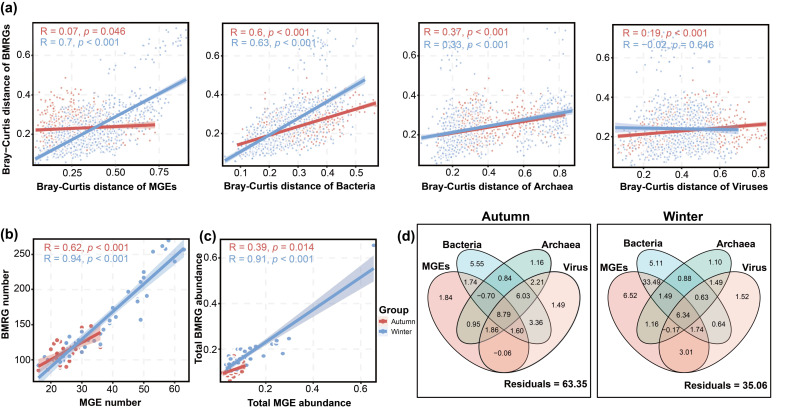
Seasonal associations among BMRGs, MGEs, and microbiomes. (**a**) Linear fitting of Bray–Curtis distances comparing BMRGs with microbiomes and MGEs across seasons. (**b**) Linear regression between the detected numbers of BMRGs and MGEs across seasons. (**c**) Linear regression showing the relationship between total BMRG and MGE abundances across seasons. (**d**) VPA revealing the relative importance of MGEs and microbial groups in explaining seasonal BMRG variation.

The associations between BMRGs and biotic factors showed clear seasonal differences (Figure 3a). Archaeal and bacterial communities were significantly associated with BMRG community structures in both autumn and winter (*p* < 0.001), whereas the association between MGEs and BMRGs increased from a marginal relationship in autumn (*p* = 0.046) to a highly significant relationship in winter (*p* < 0.001). Viral communities displayed distinct seasonal shifts in their association with BMRGs; while a significant positive correlation was observed in autumn (R = 0.19), this association completely disappeared in winter (*p* > 0.05). As illustrated in Figure 3b,c, both the number and abundance of BMRGs were significantly positively associated with those of MGEs in autumn and winter (*p* < 0.05). Notably, stronger associations were observed in winter, with R values increasing from 0.62 to 0.94 for number and from 0.39 to 0.91 for abundance.

VPA further evaluated the relative contributions of MGEs and microbial communities to the compositional variation of BMRGs. The total explained variation in VPA increased from 36.65% in autumn to 64.94% in winter (Figure 3d). The results showed that the independent contribution of MGEs to BMRG variation increased from 1.84% in autumn to 6.52% in winter. The shared contribution of MGEs and microbial hosts (especially bacteria) also increased markedly. In autumn, the shared contribution of MGEs and bacteria was only 1.74%, whereas in winter it rose sharply to 33.49%. Bacteria maintained a relatively high independent contribution across both seasons (5.55% in autumn and 5.11% in winter), indicating that they serve as important biological carriers of BMRGs.

### 3.3. Co-Occurrence Patterns of BMRGs, MGEs, and Microbiome

**Figure 4:**
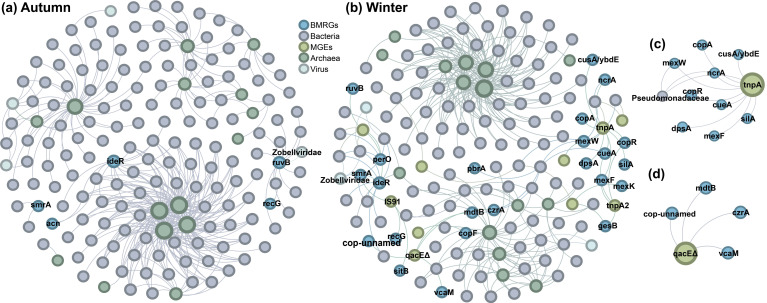
Co-occurrence networks across seasons. (**a**) Network showing interactions among BMRGs, MGEs, and family-level microbial taxa during autumn. (**b**) Network showing interactions among BMRGs, MGEs, and family-level microbial taxa during winter. (**c**) Zoomed-in subnetwork showing the direct associations of *tnpA* with connected BMRGs, MGEs and microbial taxa during winter. (**d**) Zoomed-in subnetwork showing the direct associations of *qacEΔ* with connected BMRGs, MGEs and microbial taxa during winter. Node color designates biological classifications, while node size corresponds to the degree of connectivity.

The co-occurrence network revealed the associations among BMRGs, MGEs, and microbial families during the autumn and winter seasons (Figure 4a,b). In autumn, the network consisted of 171 nodes and 368 edges with a modularity index of 0.513. Five BMRGs (*acn*, *recG*, *ruvB*, *smrA*, and *ideR*) formed 32 strong positive associations. Rather than being evenly distributed across domains, these genes exhibited clear lineage-specific patterns, co-occurring predominantly with distinct bacterial groups through 31 edges and only a single viral family. The *ideR* gene was the most connected node with 15 connections, primarily associated with Actinobacteria (e.g., *Microbacteriaceae*). Meanwhile, *ruvB* formed 12 connections and was mainly linked to Bacteroidetes. The association between *ruvB* and the *Zobellviridae* phage family represented the only BMRG-virus connection in the autumn network. In addition, no significant associations were observed between BMRGs and MGEs during this season.

The winter co-occurrence network consisted of 156 nodes and 306 edges, and its modularity index was 0.631. Compared with autumn, the winter network revealed a more extensive and integrated BMRG profile, encompassing 23 distinct resistance genes such as *ideR*, *mexW*, *smrA*, and *cueA*. Among these, nine BMRGs maintained direct taxonomic associations with 12 bacterial families and the *Zobellviridae* viral family. The most notable feature was the extensive linkage between BMRGs and MGEs. Specifically, 22 of the 23 BMRGs were statistically associated with MGEs such as *tnpA* transposases and *IS91* insertion sequences. The transposase *tnpA* was the most central MGE, linking nine BMRGs, followed by *tnpA2* and *qacEΔ*, each connecting four BMRGs. In total, 27 connections between BMRGs and MGEs were identified in winter, representing a sharp contrast to their complete absence in autumn.

### 3.4. The Influence of Environmental Factors on the Assembly of BMRGs

**Figure 5:**
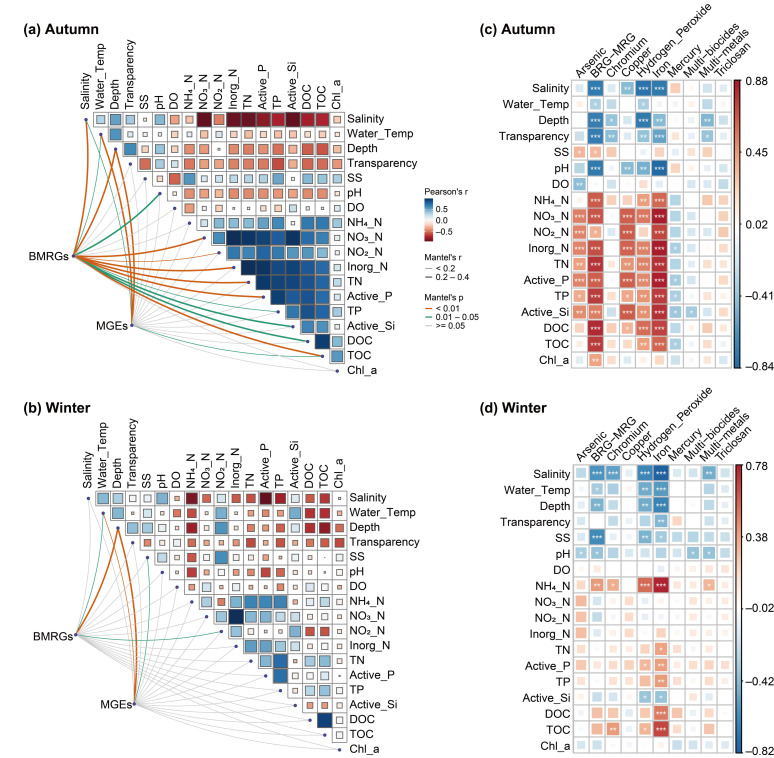
Environmental drivers of BMRG variation. (**a**) Mantel tests evaluating the relationships between environmental factors and the community structures of BMRGs and MGEs during autumn. (**b**) Mantel tests evaluating the relationships between environmental factors and the community structures of BMRGs and MGEs during winter. (**c**) Heatmaps displaying Spearman correlations between environmental variables and the 10 most prevalent BMRG types in autumn. (**d**) Heatmaps displaying Spearman correlations between environmental variables and the 10 most prevalent BMRG types in winter. *, **, and ***, represent the *p*-value < 0.05, 0.01, and 0.001, respectively.

In autumn, Mantel test results revealed that BMRGs were significantly correlated with most environmental factors (Figure 5a). Specifically, salinity, depth, Active_P, NO_3_-N, NO_2_-N, Inorg-N, TN, and TOC showed strong and highly significant associations with BMRGs (*p* < 0.01), while water temperature, pH, TP, Active_Si, and DOC were also significantly correlated with BMRGs (0.01 < *p* < 0.05). MGEs exhibited highly significant correlations with depth and water temperature (*p* < 0.01), and significant correlations with salinity and TOC (0.01 < *p* < 0.05). In contrast, fewer significant associations were observed in winter (Figure 5b). Both BMRGs and MGEs showed significant correlations with depth and water temperature (*p* < 0.05), while additional associations were detected between BMRGs and NO_2_-N and between MGEs and suspended solids (SS).

The Spearman correlation analysis further revealed that the spatial distribution of BMRGs in the Beibu Gulf was significantly influenced by environmental factors, with clear seasonal differences between autumn and winter. In autumn, BMRG distribution was primarily associated with nutrient and organic matter levels (Figure 5c). In winter, the number of significant environmental associations decreased markedly. Ammonium emerged as the dominant positive driver, followed by organic carbon, whereas salinity and depth exerted negative effects on BMRG abundance (Figure 5d).

### 3.5. Seasonal Variation in the Driving Factors and Dissemination Patterns of BMRGs

**Figure 6:**
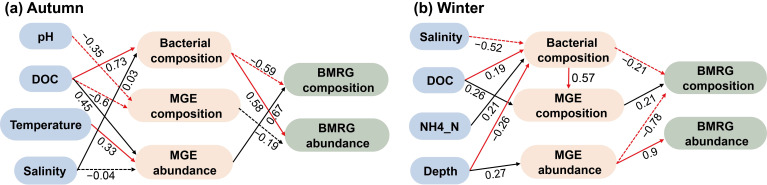
Path modeling of BMRG assembly. (**a**) PLS-PM illustrating the influence of environmental factors, microbial communities, and MGEs on BMRG assembly in autumn. (**b**) PLS-PM illustrating the influence of environmental factors, microbial communities, and MGEs on BMRG assembly in winter. Solid lines indicate positive correlations, whereas dashed lines represent negative ones. Significant paths (*p* < 0.05) are highlighted in red, and non-significant paths in black. Arrow-adjacent numbers denote path coefficients.

PLS-PM analysis was conducted to elucidate the mechanisms underlying BMRG assembly in autumn and winter. The autumn model showed a goodness-of-fit of 0.646, indicating good explanatory performance (Figure 6a). Environmental and biological factors jointly explained 50.0% of the variation in the BMRG community composition and 49.3% of the variation in total BMRG abundance. In autumn, the distribution of BMRGs was primarily influenced by the bacterial community. DOC acted as a key environmental factor, indirectly regulating BMRG composition and abundance by shaping bacterial community structure.

The winter model showed a goodness-of-fit of 0.585 (Figure 6b). Compared with autumn, the explanatory power of the model increased markedly in winter, from 50.0% to 86.4% for BMRG community composition and from 49.3% to 83.3% for total BMRG abundance (Figure 6). During winter, the contribution of MGEs to BMRG variation increased markedly, and MGE abundance showed a strong association with BMRG composition and total BMRG abundance. Meanwhile, salinity and depth also contributed to the regulation of the bacterial community and thereby indirectly influenced BMRG dynamics.

## 4. Discussion

### 4.1. Seasonal Dynamics and Persistence of BMRGs

Our investigation revealed a widespread occurrence of BMRGs in the Beibu Gulf, in which multi-biocide resistance genes showed the greatest diversity, whereas BRG-MRGs exhibited the highest abundance. These results align with previous observations in which multi-metal, biocide, and combined resistance categories exhibited the greatest subtype diversity in aquatic environments [4,5] and wastewater treatment systems [44]. The remarkable diversity of multi-biocide resistance genes may be associated with their transport-system superfamilies such as efflux pumps, which can not only resist toxic substances but also play important roles in various physiological processes [2,22]. Conversely, the high abundance of BRG-MRGs may be attributed to the persistent co-selection pressure exerted by non-degradable heavy metals [19,20]. The observed winter enrichment of BMRGs was broadly consistent with previous ARG-focused metagenomic research in the same subtropical coastal ecosystem, where ARGs and MGEs also showed clear seasonal dynamics [23]. However, this study extends previous work by demonstrating that BMRGs also exhibit season-dependent diversity, abundance, and MGE-associated dissemination patterns, highlighting additional biocide- and metal-associated co-selection risks.

The higher abundance and diversity of BMRGs in winter relative to autumn may reflect seasonal differences in selective pressures. In autumn, the lower BMRG abundance may indicate reduced benefits of maintaining non-essential resistance genes because of their associated metabolic costs [45]. In contrast, the enrichment of BMRGs in winter may suggest that resistance systems such as efflux pumps provide adaptive advantages under seasonal environmental stress, particularly through their roles in detoxification and cellular stress responses [46]. Furthermore, the robust persistence of BMRGs is exacerbated by intrinsic genetic stabilization mechanisms. Specifically, plasmids carrying BMRGs frequently harbor toxin-antitoxin (TA) systems [19] that eliminate plasmid-free daughter cells via post-segregational killing [47]. Consequently, these BMRG reservoirs achieve extraordinary longevity, maintaining their co-selection potential even under fluctuating environmental pressures [19]. These findings highlight the imperative to incorporate BMRGs into coastal environmental monitoring frameworks as a high-priority indicator of long-term ecological risk.

### 4.2. Environmental Regulation of BMRG Dynamics

The distribution of BMRGs in the Beibu Gulf was associated with the combined influences of nutrient availability, organic matter, and physicochemical gradients, particularly salinity and depth. Rather than acting independently, these variables may reflect linked environmental gradients from shallow nearshore waters to deeper offshore waters. In autumn, inorganic nitrogen (Inorg-N and NO_3_-N) and DOC were strongly positively correlated with major BMRGs, whereas NH_4_-N emerged as the primary nutrient factor associated with BMRGs in winter. Previous studies have demonstrated that nitrogen-related variables are important determinants shaping the composition and distribution of ARGs in coastal sediments [48]. Elevated nutrient levels are commonly linked to intensified terrestrial inputs, including sewage discharge and aquaculture effluents, which have been recognized as major contributors to resistance gene enrichment in estuarine and coastal ecosystems [3].

Concurrently, salinity and water depth acted as crucial ecological filters shaping the spatial distribution of BMRGs. Salinity consistently emerged as the dominant negative factor across both seasons. PLS-PM analysis indicated that salinity was mainly associated with BMRG dynamics through its relationship with bacterial community structure, rather than through a direct inhibitory effect on BMRGs. As an important ecological filter, salinity can shape microbial community composition and may thereby indirectly influence the distribution of resistance genes [49,50]. Furthermore, depth showed a significant negative correlation with BMRG abundance, indicating that resistance genes were predominantly enriched in shallower nearshore areas subjected to more direct human pressure [5]. Taken together, nutrient enrichment, salinity gradients, and water depth indicate that BMRG enrichment in the Beibu Gulf is closely linked to nearshore environmental gradients. These findings suggest that coastal monitoring should focus more on shallow nearshore areas with stronger nutrient accumulation, and that controlling land-based nutrient and pollutant inputs may help reduce the spread of BMRGs in subtropical coastal waters [51,52].

### 4.3. Seasonal Shift in BMRG-Associated Microbial and MGE Patterns

The integrated analysis suggests a seasonal shift in the dominant associations contributing to BMRG assembly, with stronger signals related to host community structure in autumn and stronger signals related to MGE-associated dissemination in winter. However, this seasonal pattern should not be interpreted as a strict alternative between bacterial communities and MGEs, because MGEs are genetic elements associated with microbial genomes or extrachromosomal replicons [53]. Accordingly, the fractions related to MGEs and bacteria should be interpreted as interconnected rather than fully independent ecological alternatives. In autumn, BMRG patterns were mainly associated with specific bacterial groups, such as Actinobacteria for *ideR* and Bacteroidetes for *ruvB*. The single BMRG-virus association involving *ruvB* and *Zobellviridae* suggests a possible viral linkage, but its ecological significance and physical linkage require further validation. In winter, the stronger correlations between BMRGs and MGEs and the increased number of co-occurrence links between BMRGs and MGEs suggest a higher potential for dissemination associated with MGEs. Elements related to transposases, especially *tnpA*, acted as central network hubs connected with multiple BMRGs. In addition, signals related to *qacEΔ* may indicate possible links with resistance platforms associated with class 1 integrons [54,55]. Previous studies have shown that *qacEΔ1* is frequently associated with class 1 integrons and may co-occur with other resistance determinants on mobile platforms, thereby enhancing the potential for co-selection [19,54,55]. Nevertheless, these associations alone do not constitute direct evidence of physical co-localization or active horizontal transfer. Overall, winter may represent a period of elevated dissemination potential for BMRGs in the Beibu Gulf, highlighting the importance of season-specific monitoring of both BMRGs and MGEs in coastal waters.

Although this study focused on seasonal differences between autumn and winter, BMRG dynamics in coastal ecosystems are likely influenced by multiple interacting factors beyond seasonality. Moreover, while our sampling design covered representative sites in the Beibu Gulf, the results may not fully reflect the spatial and seasonal heterogeneity of the entire region because of the limited number of sampling sites and the inclusion of only two seasons. Therefore, future studies should include more estuaries, broader spatial coverage, and multi-seasonal or multi-year sampling to obtain a more comprehensive understanding of BMRG dynamics in this coastal ecosystem. Future work should also integrate spatial gradients, such as nearshore-offshore distance and water depth, with environmental variables including salinity, pH, nutrient concentrations, heavy metal and biocide levels, microbial community structure, mobile genetic elements, and anthropogenic inputs.

In this study, metagenomics was used to characterize the distribution patterns of BMRGs. Unlike high-throughput quantitative PCR (HT-qPCR), which relies on predetermined targets, this approach offers comprehensive coverage of the resistome, facilitating the discovery of diverse BMRG subtypes [56]. However, metagenomic analysis can be less sensitive than HT-qPCR in detecting low-abundance genes and is partially constrained by the current scope of reference databases such as MEGARes [56]. In addition, because the present metagenomic analysis was primarily based on read-level annotation, co-occurrence networks, VPA, and path modeling, these results should be interpreted as evidence of potential relationships rather than direct proof of physical linkage or horizontal gene transfer. Therefore, future studies should include contig-level co-localization analysis, metagenome-assembled genomes, long-read sequencing, and experimental validation to confirm the physical linkage and potential transmission pathways of BMRGs and MGEs.

## 5. Conclusions

This study revealed clear seasonal distribution patterns and potential dissemination risks of BMRGs in the Beibu Gulf. Winter samples showed higher BMRG diversity and abundance than autumn samples; multi-biocide resistance genes were the most diverse, whereas BRG-MRGs were the most abundant. Integrated analyses indicated that autumn BMRG patterns were more strongly associated with bacterial community structure and nutrient conditions, whereas winter patterns showed stronger associations with MGEs, suggesting that winter may represent a period of elevated potential dissemination risk for BMRGs in this ecosystem. These findings support seasonally targeted monitoring of BMRGs and MGEs and stronger management of land-based nutrient and pollutant inputs. Future studies should broaden spatial and temporal coverage by incorporating sediment samples and long-term monitoring, while integrating higher-resolution genomic approaches to better characterize the seasonal dynamics and environmental dissemination potential of BMRGs in coastal ecosystems.

## Data Availability

The data presented in this study are available on request from the corresponding author due to project-related restrictions requiring a temporary embargo on public release of the dataset.

## Supplementary Materials

The following supporting information can be downloaded at: https://www.mdpi.com/article/10.3390/microorganisms14071480/s1, Figure S1: Overview of numbers of detected BMRG subtypes in different samples. Figure S2: Heatmap revealing the abundance patterns of dominant BMRG subtypes in different samples.
