# Supplementary material for: Metagenomic Insights into the Seasonal Distribution and Dissemination Risks of Biocide and Metal Resistance Genes in a Subtropical Coastal Ecosystem
Source: Microorganisms. 2026 Jul 7;14(7):1480. doi: 10.3390/microorganisms14071480 (PMC13414413; doi:10.3390/microorganisms14071480)
Supplement: Supplementary file 1 [file microorganisms-14-01480-s001.zip › microorganisms-4366770-supplementary.pdf]

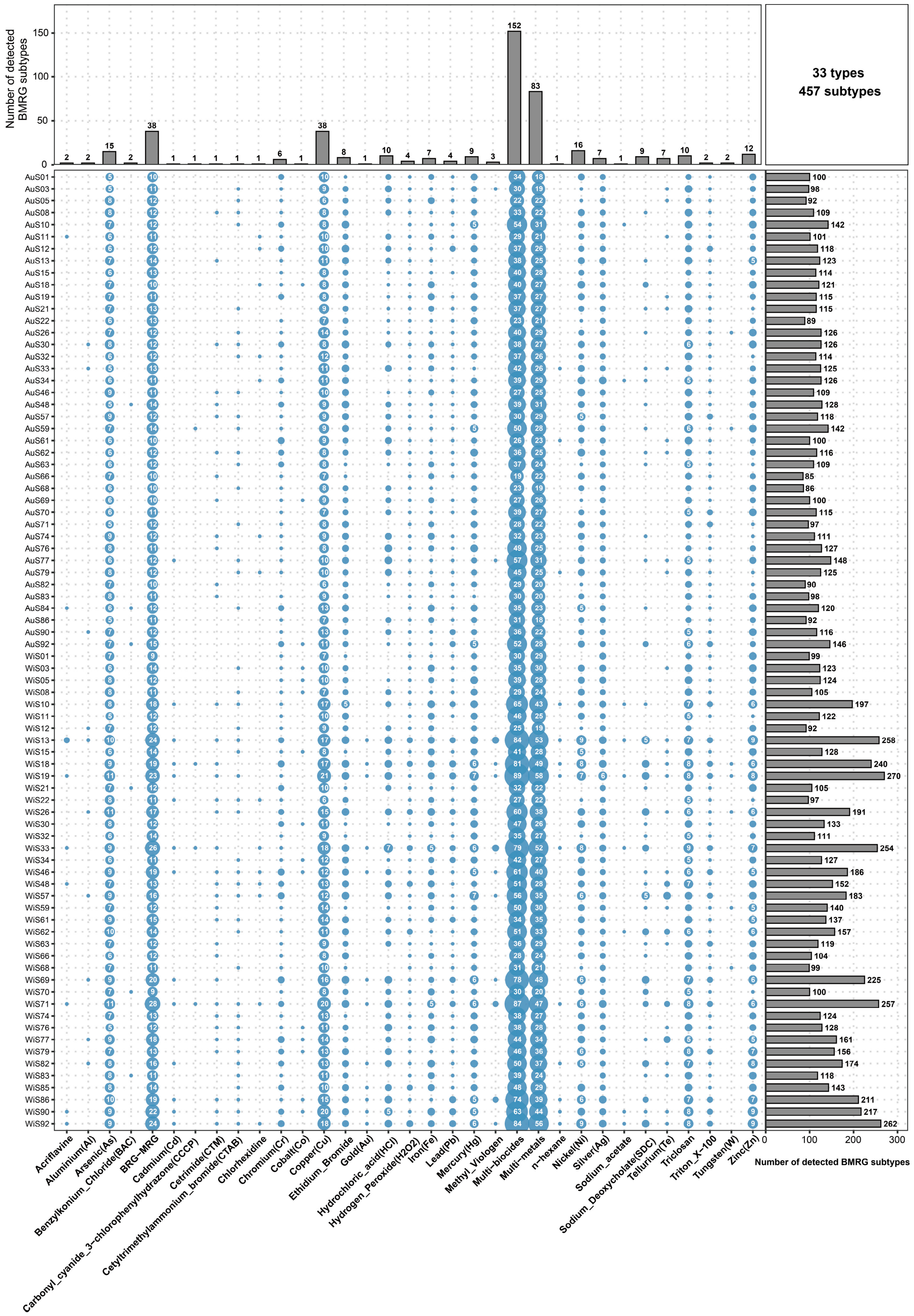

Figure S1. Overview of numbers of detected BMRG subtypes in different samples.

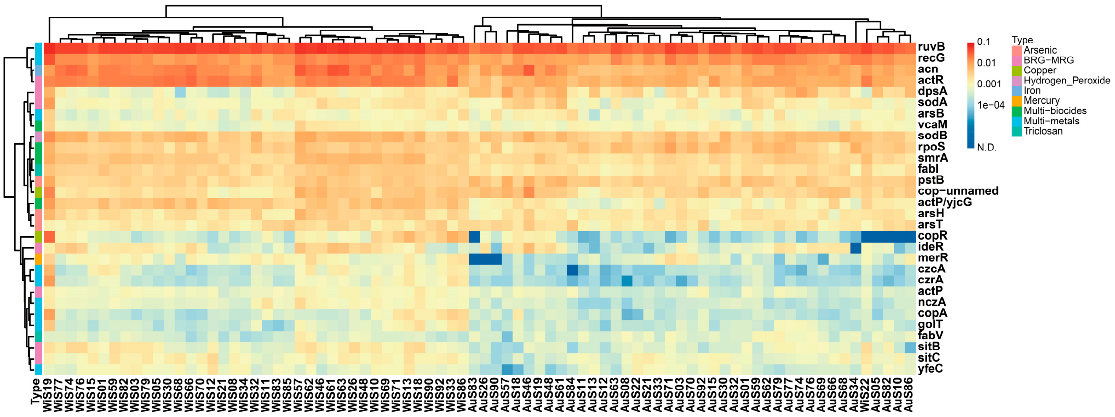

Figure S2. Heatmap revealing the abundance patterns of dominant BMRG subtypes in different samples.
